# Supplementary material for: Empirical methods for controlling false positives and estimating confidence in ChIP-Seq peaks
Source: BMC Bioinformatics. 2008 Dec 5;9:523. doi: 10.1186/1471-2105-9-523 (PMC2628906; doi:10.1186/1471-2105-9-523)
Supplement: Additional File 1 — A variety of html documents from the USeq web site detailing the available applications, their best usage, output file type descriptions, command line menus, etc. [file 1471-2105-9-523-S1.zip › USeqUserGuides/JavaDoc.zip!/JavaDoc/edu/utah/seq/analysis/package-summary.html]

edu.utah.seq.analysis


|  |  |  |
| --- | --- | --- |
| **Overview** \| **Package** \| Class \| **Use** \| **Tree** \| **Deprecated** \| **Index** \| **Help** | |  |
| **PREV PACKAGE**   **NEXT PACKAGE** | **FRAMES**    **NO FRAMES**     **All Classes** |  |


---

## Package edu.utah.seq.analysis

Primary analysis applications and helper classes for working with binary PointData, xxx.bar.zip files.

**See:**
  
          **Description**

| **Class Summary** | |
| --- | --- |
| **DefinedRegionScanSeqs** | Takes PointData and a set of defined regions, either a UCSC gene table or a bed file and scores the regions. |
| **EnrichedRegionMaker** | Collapses an array of window into and array of enriched regions based on score and max gap. |
| **PeakPicker** | Use to find the peaks within EnrichedRegions |
| **QCSeqs** | Takes PointData and generates sliding window average scores. |
| **ScanSeqs** | Takes PointData and generates sliding window scan statistics. |

## Package edu.utah.seq.analysis Description

Primary analysis applications and helper classes for working with binary PointData, xxx.bar.zip files.

---


|  |  |  |
| --- | --- | --- |
| **Overview** \| **Package** \| Class \| **Use** \| **Tree** \| **Deprecated** \| **Index** \| **Help** | |  |
| **PREV PACKAGE**   **NEXT PACKAGE** | **FRAMES**    **NO FRAMES**     **All Classes** |  |


---
